# NRG1/ERBB3/ERBB2 Axis Triggers Anchorage-Independent Growth of Basal-like/Triple-Negative Breast Cancer Cells

**DOI:** 10.3390/cancers14071603

**Published:** 2022-03-22

**Authors:** Carmen Miano, Alessandra Morselli, Francesca Pontis, Chiara Bongiovanni, Francesca Sacchi, Silvia Da Pra, Donatella Romaniello, Riccardo Tassinari, Michela Sgarzi, Elvira Pantano, Carlo Ventura, Mattia Lauriola, Gabriele D’Uva

**Affiliations:** 1 National Laboratory of Molecular Biology and Stem Cell Engineering, National Institute of Biostructures and Biosystems (INBB), 40129 Bologna, Italy; carmen.miano@unibo.it (C.M.); chiara.bongiovanni2@unibo.it (C.B.); francesca.sacchi7@unibo.it (F.S.); silvia.dapra2@unibo.it (S.D.P.); riccardo.tassinari.rt@gmail.com (R.T.); carlo.ventura@unibo.it (C.V.); 2 Centre for Applied Biomedical Research (CRBA), University of Bologna, 40138 Bologna, Italy; 3 Department of Experimental, Diagnostic and Specialty Medicine, University of Bologna, 40138 Bologna, Italy; alessandra.morselli2@studio.unibo.it (A.M.); donatella.romaniello@unibo.it (D.R.); michela.sgarzi2@unibo.it (M.S.); mattia.lauriola2@unibo.it (M.L.); 4 Scientific and Technological Pole, IRCCS MultiMedica, 20138 Milan, Italy; francesca.pontis@istitutotumori.mi.it (F.P.); elvira.pantano@multimedica.it (E.P.)

**Keywords:** basal-like breast cancer, triple-negative breast cancer, neuregulin 1, ERBB3, HER3, ERBB2, HER2, anchorage-independent growth, 3D growth, spheroids, HER2-targeting agents, pertuzumab, trastuzumab

## Abstract

**Simple Summary:**

Breast cancer is a heterogeneous disease, categorized into distinct subgroups with different clinical prognoses and treatment strategies. This study aimed to evaluate the role of ERBB3 in different molecular subtypes of breast cancers. Despite ERBB3/HER3 and its partner ERBB2/HER2 showing low expression levels in basal-like/triple-negative breast cancers, stratification of basal-like patients according to ERBB3 mRNA expression levels highlighted a correlation between higher *ERBB3* levels and shorter relapse-free patients’ survival. In vitro analyses unveiled that the activation of the NRG1/ERBB3/ERBB2 axis robustly induces anchorage-independent growth of basal-like/triple-negative breast cancer cellular models, without significant effects on cell proliferation, differentiation, and migration in adhesion. Overall, our data suggest that ERBB2/ERBB3 plays an oncogenic role in basal-like/triple-negative breast cancer patients, suggesting its neutralization as a therapeutic strategy for these breast cancer subtypes, which today have very limited treatment opportunities.

**Abstract:**

ERBB3, also known as HER3, is a tyrosine kinase transmembrane receptor of the ERBB family. Upon binding to neuregulin 1 (NRG1), ERBB3 preferentially dimerizes with HER2 (ERBB2), in turn inducing aggressive features in several cancer types. The analysis of a dataset of breast cancer patients unveiled that higher *ERBB3* mRNA expression correlates with shorter relapse-free survival in basal-like breast cancers, despite low *ERBB3* expression in this breast cancer subtype. Administration of neuregulin 1 beta (NRG1β) significantly affected neither cellular proliferation nor the basal migratory ability of basal-like/triple-negative quasi-normal MCF10A breast cells, cultured in mono-layer conditions. Furthermore, no significant regulation in cell morphology or in the expression of basal/myoepithelial and luminal markers was observed upon stimulation with NRG1β. In non-adherent conditions, NRG1β administration to MCF10A cells did not significantly influence cell survival; however, it robustly induced cell growth as spheroids (3D growth). Intriguingly, a remarkable upregulation of ERBB3 and ERBB2 protein abundance was observed in 3D compared to 2D cell cultures, and NRG1β-induced 3D cell growth was efficiently prevented by the anti-HER2 monoclonal antibody pertuzumab. Similar results were obtained by the analysis of basal-like/triple-negative breast cancer cellular models, MDA-MB-468 and MDA-MB-231 cells, in which NRG1β induced anchorage-independent cell growth that in turn was prevented or reduced by the simultaneous administration of anti-HER2 neutralizing antibodies. Finally, the ability of pertuzumab in suppressing NRG1β-induced 3D growth was also evaluated and confirmed in MCF10A engineered with HER2-overexpression. We suggest that the NRG1/ERBB3/ERBB2 pathway promotes the anchorage-independent growth of basal-like breast cancer cells. Importantly, we provide evidence that ERBB2 neutralization, in particular by pertuzumab, robustly inhibits this process. Our results pave the way towards the development of novel anticancer strategies for basal-like breast cancer patients based on the interception of the NRG1/ERBB3/ERBB2 signaling axis.

## 1. Introduction

ERBB/HER receptors (EGFR/ERBB1, ERBB2/HER2, ERBB3/HER3, and ERBB4/HER4) play major roles in the development and progression of various solid cancers, including those arising from the breast tissue (reviewed in [1,2,3]). Upon binding of specific ligands, ERBB receptors form homo- and/or hetero-dimers with other family members, and activate multiple downstream signaling cascades (reviewed in [1,2,3]). Neuregulins (NRGs) are growth factors binding to ERBB3 and/or ERBB4, predominantly inducing heterotypic interactions with ERBB2 (reviewed in [1,2,3,4]). ERBB3 is involved in tumor development and progression of different cancer types, including breast cancer (reviewed in [1,5]). In addition, ERBB3 mutations are not uncommon in many cancer types [6] (reviewed in [7]). ERBB2 is unable to bind ligands, yet it is the preferred hetero-dimerization partner for the other ERBBs, amplifying and diversifying the signaling cascades [8] (reviewed in [1,2,3]). ERBB3 and ERBB2 levels are frequently associated, especially in human breast cancers, where ERBB3/ERBB2 heterodimers function as an oncogenic driver for breast tumor cell proliferation [9] (reviewed in [1,5]). ERBB3 is detectable in preneoplastic ERBB2-overexpressing breast epithelium and is required for cell growth and malignant progression in ERBB2-driven hyperplasia [10]. The NRG1-ERBB3-ERBB2 signaling axis has been shown to induce the self-renewal and proliferation of breast tumor-initiating cells and cancer stem cells [11,12].

Breast cancer is a heterogeneous disease. For this reason, it is classified into different subtypes with distinct clinical behaviors and prognoses and different treatment strategies (reviewed in [13,14]). Therefore, the accurate classification of breast cancer subtypes is crucial for personalized disease management and for improving patient outcomes. In clinical practice, breast cancer classification driving the treatment-decision process is established on the distinction between major breast cancer subsets based on conventional immunohistochemistry (IHC) and in situ hybridization (ISH) analyses of ERBB2 (HER2), estrogen receptor alpha (ER), and progesterone receptor (PR) (reviewed in [13,14]). Based on IHC/ISH analyses, three clinical breast cancer subgroups can be identified: HER2+, hormone receptor-positive, and triple-negative. Breast cancer expressing ERBB2/HER2 are classified as HER2+, independently from the expression of ER and/or PR. Conversely, breast cancers expressing ER and/or PR, but not ERBB2/HER2, are termed “hormone receptor-positive”, “hormone responsive” or “luminal” tumors. Tumors not expressing ER, PR, and ERBB2/HER2 are called “triple-negative” (TNBC). Anti-HER2 agents are recommended for the treatment of HER2+ breast cancer patients, whereas the administration of ER antagonists is recommended to hormone receptor-positive breast cancer patients. Unfortunately, less efficient targeted treatment strategies are currently available for “triple-negative” cancer patients. Indeed, the only targeted therapy currently approved for triple-negative breast cancers is pembrolizumab (an antibody against programmed death-receptor PD-1 that provides dual ligand blockade of programmed death-ligands PD-L1 and PD-L2). However, about 1 out of 5 triple-negative breast cancer patients expresses PD-L1 protein, thus only a small subset of triple-negative patients can benefit from the combinatorial administration of pembrolizumab and chemotherapy (reviewed in [14,15]).

In the last two decades, different breast cancer molecular subtypes have been identified by gene expression data [16,17,18] (reviewed in [13,14,19]). Molecular subtypes of breast carcinomas include basal-like (BLBC), HER2-enriched, luminal A, and luminal B. In this regard, basal-like breast cancers are characterized by poor prognosis and a high rate of relapse [20,21,22] (reviewed in [23,24,25]). Notably, basal-like and triple-negative breast cancers exhibit a high degree of gene expression profile overlap, with ∼80% of BLBCs being ER-negative and HER2-negative, and 50–75% of TNBCs displaying a basal-like phenotype [20,21,22] (reviewed in [23,24,25]). Unfortunately, similar to triple-negative breast cancers, basal-like cancer subtypes have very limited treatment opportunities to date.

In this study, we investigated the potential role of NRG1/ERBB3/ERBB2 signaling in different clinical and molecular subtypes of breast cancers, by combining metanalysis on patients’ survival and gene expression, and by exploring in vitro potential effects induced by the activation of this pathway.

## 2. Materials and Methods

### 2.1. Bioinformatic Analysis of Breast Cancer Patients’ Data

Evaluation of mRNA expression of genes of interest in normal breast tissue and/or breast cancers stratified for molecular and clinical subtypes was conducted by “bc-GenExMiner” [26] (http://bcgenex.ico.unicancer.fr/, accessed on 9 March 2022) and “UALCAN” [27] (http://ualcan.path.uab.edu/, accessed on 9 March 2022), respectively. Evaluation of mRNA expression of genes of interest in breast cancer cell lines stratified for molecular and clinical subtypes was conducted by “Gene expression-based Outcome for Breast cancer Online” (GOBO) [28] (http://co.bmc.lu.se/gobo/gsa.pl, accessed on 9 March 2022). GOBO gene set expression analysis in breast cancer cell lines (GSA-Cell line) includes mRNA expression data across 51 breast cancer cell lines [29].

Analyses of relapse-free survival (RFS) of breast cancer patients were conducted by the Kaplan-Meier (KM) plotter online database [30] (http://kmplot.com/, accessed on 9 March 2022). KM plotter sources for the databases include GEO, EGA, and TCGA. Patients belonging to specific breast cancer molecular subtypes were stratified into two groups according to their expression levels of *ERBB3* mRNA, and relapse-free survival (RFS) after tumor resection was calculated by the Kaplan–Meier curve and log-rank test during a follow-up period of 120 months. The best performing threshold between the lower and upper quartiles was used as a cutoff. The results were shown in the Kaplan–Meier survival plots. *p*-value, hazard ratio (HR), and false discovery rate (FDR) are provided. *p*-value < 0.05 by using the log-rank test was regarded as statistically significant. Hazard ratios were used to estimate the effect for time-to-event end points, such as relapse-free survival. A hazard ratio of 1 means lack of association, a hazard ratio greater than 1 suggests an increased risk, and a hazard ratio below 1 suggests a smaller risk.

### 2.2. Cell Cultures

In vitro experiments have been conducted in breast cell line MCF10A and in previously generated HER2-overexpressing MCF10A (MCF10A-HER2) and their control (MCF10A-ctrl) cells [31,32]. MCF10A cells and derivative lines (MCF10A-HER2 and MCF10A-ctrl) were kindly provided by Prof. Yarden’s laboratory (Weizmann Institute of Science) and were cultured in DMEM-F12 medium containing 5% of horse serum (HS), 1% penicillin/streptomycin, 1% L-glutamine, 0.5 µg/mL of hydrocortisone, 100 ng/mL of cholera toxin, 0.5 µg/mL of insulin and 10 ng/mL of EGF (hereafter “full medium”). Further experiments have been performed on breast cancer cell lines MDA-MB-468 and MDA-MB-231. Both the cell lines were cultured in RPMI-1640 medium containing 10% fetal bovine serum (FBS). The cells have been grown in 10 cm plastic Petri dishes and incubated at 37 °C in a humidified atmosphere of 5% CO_2_/air.

### 2.3. Proliferation and Random/Directional Migration Analysis in Monolayer Conditions

Proliferation and migration analyses on MCF10A cells were performed using a Livecyte^TM^ technology (Phase Focus, Sheffield, UK). Cells were treated with neuregulin 1β (10 ng/mL), trastuzumab (10 µg/mL), and pertuzumab (10 µg/mL), alone and in combination, before the start of the experiment. Trastuzumab or pertuzumab was added at least 30 min before adding NRG1β. Images were acquired every 60 min for 72 h with a 10× objective, at 37 °C and 5% CO_2_/air. Data were analyzed using Cell Analysis Toolbox software (Phase Focus, Sheffield, UK). Cell proliferation was determined by Livecyte software by counting the number of cells in each frame. Cell motility was evaluated by measuring cell velocity, calculated as the change in position in each frame. The degree of directional versus random migration was estimated by calculating cell displacement and the cell confinement ratio. Indeed, these two parameters represent the distance a cell migrates relative to its point of origin and consider the degree to which a cell meanders from its starting and end points. In particular, cell displacement shows the position of cells and their trajectories over time, relative to their point of origin. Confinement ratio is the ratio of the length of the direct path between the initial and the final position over the total track length. To circumvent the problem of dependency on the cell track duration, the confinement ratio was multiplied by the square root of time. Morphological and morphometrical analyses were performed by Livecyte software calculating the area of cells in each frame, the sphericity, measuring how close to a sphere is a cell in each frame, and the length to width ratio, deriving from the calculation of how round versus elongated a cell is in each frame.

Then, 50,000 MDA-MB-468 and MDA-MB-231 cells/well were seeded into a six-well plate in full medium. Treatments with neuregulin 1β (10 ng/mL) were added the day after seeding. After three days of treatments, cells were trypsinized and manually counted using the Neubauer Chamber. Representative pictures were acquired by using EVOS™ M5000 Imaging System at 20× magnification.

### 2.4. Anchorage-Independent Growth Assay

To assess the ability of cells to grow in anchorage-independent conditions, a growth assay was carried out in ultra-low attachment six-well plates. For each well, 10,000 cells were seeded in 2 mL of complete medium. Treatments with neuregulin 1β, trastuzumab, and pertuzumab, alone and in combination, were added immediately and repeated every three days. After two weeks of treatment, pictures of the wells were collected using an inverted microscope at 4× magnification. Spheroids from each condition were counted and areas were measured using ImageJ software.

### 2.5. RNA Isolation and Real Time PCR

Next, 100,000 MCF10A cells were seeded in six-well plates in full medium. Treatments were added and repeated every 3–4 days. After 9 days, cultured cells were washed, trypsinized, and collected. Total RNA was extracted with a NucleoSpin RNA II kit (Macherey Nagel), following the manufacturer’s protocol specifications. Total RNA quantity and quality were determined using a NanoDrop spectrophotometer (N1000, Thermo Fisher Scientific, Waltham, MA, USA). RNA reverse transcription to double-stranded cDNA was performed using SuperScript™ VILO™ cDNA Synthesis Kit (Invitrogen™, Waltham, MA, USA), according to the manufacturer’s protocol, followed by incubation in the thermocycler for the reaction. Real Time (rt)-PCR was performed with a QuantStudio 6 Flex instrument (Applied Biosystems, Waltham, MA, USA) by using Fast SYBR Green PCR Master Mix (Applied Biosystems, Waltham, MA, USA), and analysis was conducted on QuantStudio 6 Flex Real-Time PCR System software. Oligonucleotide sequences of analyzed genes, namely KRT5, KRT14, TP63, KRT8, MUC1, and HPRT1 (Sigma-Aldrich, Saint Louis, MO, USA), are listed in Supplementary Table S1. Relative quantification was performed using HPRT1 gene as a loading control. DDCT was calculated and data of each gene were analyzed using a 2^−^^DDCT^ method and reported as mean fold change.

### 2.6. Analysis of Cell Cycle Activity on Cultured Cells by Immunofluorescence Evaluation of KI67

Then, 2000 MCF10A cells/well were seeded into a 96-well plate in full medium. Treatments with neuregulin 1β, trastuzumab, and pertuzumab, alone and in combination, were added immediately and repeated every 3 days. After 9 days of treatments, cells were fixed in 4% paraformaldehyde (PFA) for 20 min. Samples were washed and permeabilized with 0.5% Triton-X100 (Sigma-Aldrich, Saint Louis, MO, USA) in PBS for 5 min at room temperature. Then, to avoid the non-specific binding of the antibodies, samples were blocked with PBS supplemented with 5% bovine serum albumin (BSA) (Sigma-Aldrich, Saint Louis, MO, USA) and 0.1% Triton-X100 for 1 h at room temperature. To analyze cell-cycle re-entry, samples were incubated overnight at 4° C with anti-KI67 primary antibody (1:100, ab16667, Abcam, Cambridge, UK) diluted in PBS supplemented with 3% BSA and 0.1% Triton-X100. After primary antibody incubation, samples were washed 3 times in PBS and incubated at room temperature for 1 h with the fluorescent secondary antibody anti-rabbit 594 (1:200, AlexaFluor 111-585-003, Jackson), diluted in PBS supplemented with 1% BSA and 0.1% Triton-X100. Afterward, samples were washed three times in PBS. Then, for nuclei visualization, samples were stained for 15 min at room temperature with DAPI (4’,6-diamidino-2-phenylindole dihydrochloride, Sigma-Aldrich, Saint Louis, MO, USA), diluted in PBS (1:1000). Samples were then washed twice more in PBS. Finally, pictures were acquired by using a Zeiss widefield microscope (Axio Observer A1) or an ArrayScan XTI widefield microscope (Thermo Fisher Scientific, Waltham, MA, USA). The number of KI-67 positive cells was quantified using ImageJ software.

### 2.7. Protein Extraction and Evaluation by Western Blot Analysis

MCF10A cells were seeded on 6 cm plastic Petri dishes (500,000 cells) and cultured as a monolayer. After 4 days of culture, they were washed, trypsinized, and collected. MCF10A cells grown in anchorage-independent conditions (10,000 cells/well) were washed and collected after 14 days of culture. Both adherent and non-adherent MCF10A cells were resuspended in RIPA buffer supplemented with a protease inhibitor cocktail (P8340, Sigma-Aldrich, Saint Louis, MO, USA, 1:100) and Na_3_VO_4_ (1 mM). Then, 65 μg of protein extracts were resolved by sodium dodecyl sulfate (SDS)-polyacrylamide gel electrophoresis and transferred to a nitrocellulose membrane (AmershamTM ProtranTM Premium 0.45 μm 300 mm × 4 m). Next, the membrane was blocked for 60 min using TBS-T (0.1% Tween-20) supplemented by 3% BSA (Sigma-Aldrich, Saint Louis, MO, USA), and incubated overnight (4 °C) with the following primary antibodies: anti-ERBB2 monoclonal antibody (1:1000 dilution; #4290 Cell Signaling Technology, Inc., Danvers, MA, USA), anti-ERBB3 (1:1000 dilution; #4754 Cell Signaling Technology, Inc., Danvers, MA, USA), anti-GAPDH (1:1000 dilution; #G9545 Sigma-Aldrich, Saint Louis, MO, USA). For protein detection, the membrane was incubated with anti-rabbit horseradish peroxidase-labeled secondary antibody (Dako EnVision+ System- HRP Labelled Polymer) followed by a chemiluminescent reaction (Clarity Western ECL Substrate, Bio-Rad). Signals and images were acquired by Chemi Doc™ XRS 2015 (Bio-Rad Laboratories, Hercules, CA, USA), and densitometric analysis was performed using Image Lab software (version 5.2.1; Bio-Rad Laboratories, Hercules, CA, USA). The original western blot figures can be found in Figure S9.

### 2.8. Cell Survival Analysis by Flow Cytometry

MCF10A cells were seeded on ultra-low attachment 6-well plates. For each well, 100,000 cells were seeded in 2 mL of full medium. Treatments with neuregulin 1β were added immediately. After 24, 48, and 72 h, respectively, cells were harvested and washed twice in PBS. Annexin V-APC was added to the cell suspension for 15 min, according to the manufacturer’s protocol (Biolegend, San Diego, CA, USA). After Annexin V staining, cells were resuspended in Annexin binding buffer and propidium iodide (PI) was added. Cells were analyzed by CytoFLEX Flow cytometer through CytExpert software. Cells positive for Annexin V-APC were identified as early apoptotic cells, whereas cells positive for both Annexin V-APC and propidium iodide were considered as late apoptotic/necrotic cells. A minimum of 10,000 events were recorded for each sample.

### 2.9. Statistical Analysis

Statistical analyses were performed with GraphPad Prism 8 software. Whenever normality could be assumed, statistical differences between group means were determined using the two-sided Student’s *t*-test or analysis of variance (ANOVA) followed by Tukey’s or Sidak’s test, as specified in the figure legends. *p*-value < 0.05 was considered to represent a statistically significant difference. In all panels, numerical data are expressed as mean ± standard error of the mean (s.e.m.); results are marked with one asterisk (*) if *p* < 0.05, two (**) if *p* < 0.01, three (***) if *p* < 0.001, and four (****) if *p* < 0.0001. The sample size was determined considering the variability observed in preliminary and similar experiments.

## 3. Results

### 3.1. mRNA Expression Levels of ERBB3 Correlate with Shorter Relapse-Free Survival in Basal-like Breast Cancer Patients

**Figure 1:**
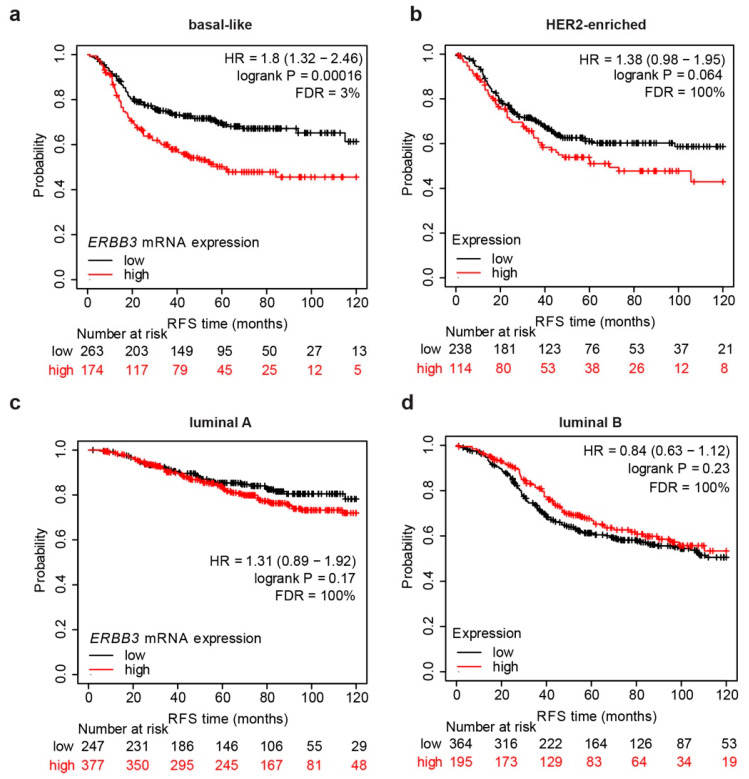
Relapse-free survival of breast cancer patients stratified for molecular subtypes and *ERBB3* mRNA levels. (**a**–**d**) Relapse-free survival (RFS) of (**a**) basal-like (*n* = 437), (**b**) HER2-enriched (*n* = 352), (**c**) luminal A (*n* = 624), and (**d**) luminal B (*n* = 559) breast cancer patients stratified into two groups according to *ERBB3* mRNA levels. Stratification in molecular subtypes was obtained by a prediction analysis of microarray 50 (PAM50). Data on normal-like subgroup are not provided due to a low number of patients. *p*-value, hazard ratio (HR), and false discovery rate (FDR) are also provided.

We started our investigation by evaluating the correlation between *ERBB3* abundance and the relapse-free survival (RFS), namely the time that the patient survives without any cancer sign after primary treatment. To this end, breast cancer patients were divided into different molecular subtypes and stratified into two groups according to the expression levels of *ERBB3* mRNA. Breast cancer patients’ stratification in molecular subtype was performed by a prediction analysis of microarray 50 (PAM50) [33], a 50-gene signature that classifies breast cancer into five subgroups: basal-like, HER2-enriched, luminal A, luminal B, and normal-like. RFS after tumor resection was calculated during a follow-up period of 10 years. Surprisingly, we found that higher *ERBB3* mRNA levels predict a significantly shorter RFS in basal-like breast cancer patients (Figure 1a), whereas no significant correlation was observed in other breast cancer subtypes (Figure 1b–d). The correlation between increased *ERBB3* mRNA levels and lower RFS was quite substantial. Indeed, the group of patients with higher *ERBB3* mRNA levels compared with lower *ERBB3* mRNA levels reported an 80% increased risk (hazard ratio = 1.8). These data suggest an oncogenic role of ERBB3 in basal-like breast cancer patients.

**Figure 2:**
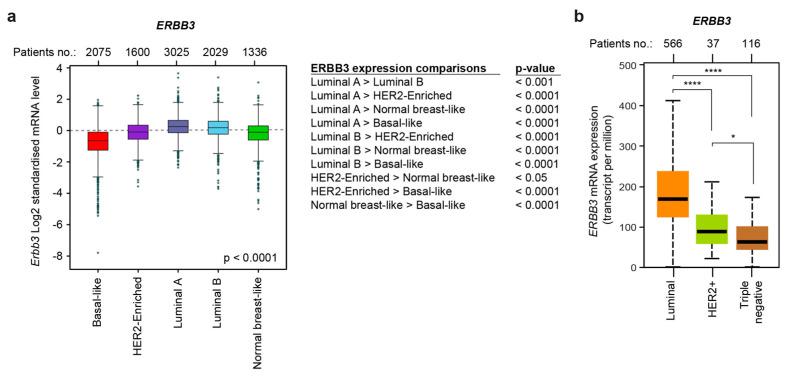
Basal-like/triple-negative breast cancers express low *ERBB3* mRNA levels compared to other breast cancer subtypes. (**a**) Box plot of *ERBB3* gene expression in breast cancer patients stratified for molecular subtypes (PAM50, *n* = 10,065 patients); (**b**) box plot of *ERBB3* gene expression in breast cancer patients stratified for clinical subtypes (*n* = 719 patients); *p*-values are provided; * *p* < 0.05, ** *p* < 0.01, *** *p* < 0.001, and **** *p* < 0.0001.

We continued our investigation by evaluating the mRNA expression levels of the ERBB3 receptor in different molecular subtypes of breast cancer patients. Our data show that the average expression levels of *ERBB3* mRNA modestly diverge in different breast molecular subtypes (Figure 2a). In detail, luminal A and B breast cancer patients displayed the highest *ERBB3* mRNA levels, HER2-enriched and normal-like breast cancer patients showed intermediate levels and basal-like breast cancer patients exhibited the lowest levels. Similarly, the evaluation of the mRNA expression levels of *ERBB3* in breast cancer specimens stratified for clinical subtypes demonstrated that the luminal, HER2+, and triple-negative subgroup display the highest, intermediate and lowest levels, respectively (Figure 2b). Furthermore, the analysis of the ERBB3 ligand NRG1 unveiled very low levels in breast cancer tissue compared to normal specimens, with minimal differences among clinical and molecular breast cancer subtypes (Supplementary Figure S1a,b). These data suggest that ERBB3 exerts an oncogenic role in basal-like/triple-negative tumors, despite the low expression levels of ERBB3 receptor in these breast cancer subtypes.

### 3.2. Neuregulin 1β (NRG1β) Does Not Significantly Induce Proliferation or Motility in Basal-like/Triple-Negative Breast Cells Cultured in Monolayer Conditions

Basal-like breast cancer is an aggressive molecular subtype characterized by a molecular signature similar to that of basal myoepithelial cells, and nowadays the treatment options are very few. To evaluate a potential role for ERBB3 in basal-like breast cancer cell aggressiveness, we decided to perform in vitro experiments upon activation of this receptor. To select an appropriate cellular model, we analyzed the levels of *ERBB3* mRNA in 51 breast cell lines, which were previously characterized and divided into luminal and basal-like (basal A and basal B) subgroups [29]. Basal-like cell lines, in particular within the basal B subgroup, showed lower *ERBB3* mRNA levels compared to luminal cell lines (Supplementary Figure S2a,b), mirroring the observation in basal-like breast cancer patients. In accordance, triple-negative breast cancer exhibits low expression levels of *ERBB3* compared to the other clinical subtypes (Supplementary Figure S2c). Furthermore, a modest increase in *NRG1* mRNA levels was observed in the basal-B subgroup (Supplementary Figure S2d,e), while the stratification for clinical breast cancer subtypes highlighted a high variability in the triple-negative subgroup without significant differences with the other subtypes (Supplementary Figure S2f). Among basal-like cell lines, we then selected MCF10A cells as the cellular model. These cells were derived from benign breast tissue spontaneously immortalized without defined factors [34]. Thus, the employment of MCF10A cells as a cellular model avoids the interference of multiple aberrations usually occurring in cancer cells. In addition, MCF10A cells are a widely employed and accepted model to study the mechanistic contribution of different ERBB family members [31,32,35,36]. MCF10A cells, as most basal B cell lines, express very low levels of ERBB2 (Supplementary Figure S3a–c), ER-alpha (Supplementary Figure S3d–f), and PR (Supplementary Figure S3g–i), thus they are also considered “triple-negative” [37] (reviewed in [38]).

**Figure 3:**
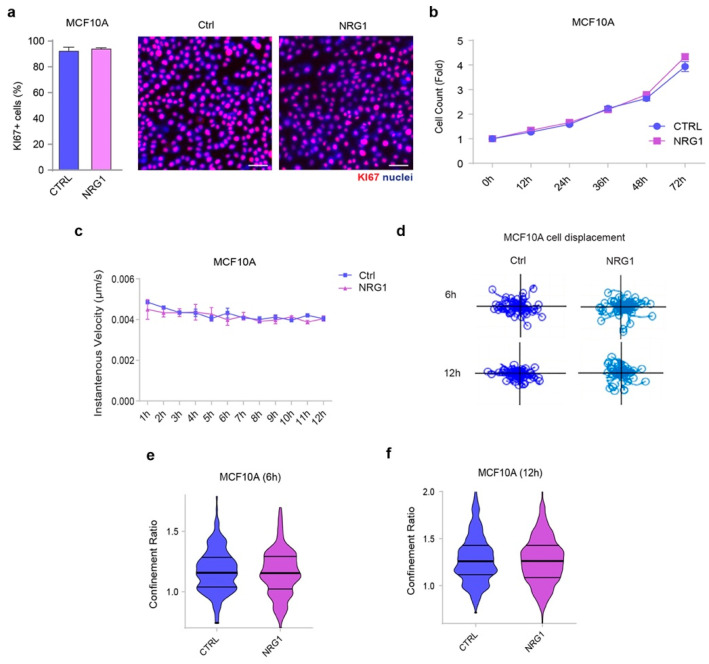
Proliferation and migration of basal-like/triple-negative breast cells cultured in monolayer conditions are not affected by the administration of the growth factor NRG1β. (**a**) Immunofluorescence analysis of KI67 for the evaluation of cells in the active phases of the cell cycle. MCF10A cells were cultured in vitro and treated with/without NRG1β (10 ng/mL) for seven days. Subsequently, they were fixed and stained with an anti-KI67 antibody and DAPI (for nuclear staining) and examined with an inverted fluorescent microscope. Photoshop software was used to quantify KI67 positive cells of three different images taken for each sample. Representative pictures are provided; scale bar, 10 μm. The histograms show the analysis of positively stained cells (means ± SEM) for MCF10A control and NRG1-treated cells, respectively. (**b**) Cell count analysis over time by Livecyte technology. MCF10A were cultured in vitro and treated with/without NRG1β (10 ng/mL) and monitored up to 72 h. (**c**) Cell speed analysis over time by Livecyte technology of MCF10A cells treated with/without NRG1β (10 ng/mL). Cell velocity has been detected every hour up to 12 h. (**d**) MCF10A cell displacement analysis by Livecyte technology. Representative track plots of MCF10A treated with/without NRG1β (10 ng/mL) up to 6 and 12 h are provided. (**e**,**f**) Confinement ratio analysis of MCF10A cells by Livecyte technology. Violin plots of MCF10A treated with/without NRG1β (10 ng/mL) for 6 and 12 h.

To evaluate a potential role for ERBB3 signaling in basal-like/triple-negative breast cells, we employed the ERBB3 ligand neuregulin 1β (NRG1β). Firstly, MCF10A cells were treated with medium containing NRG1β (10 ng/mL) or with control medium for seven days, then fixed and analyzed for cell cycle activity by immunostaining of KI67 protein. Indeed, nuclear KI67 immunoreactivity is detectable during all the active phases of the cell cycle (G1, S, G2, and M phases), but not in resting cells (G0 phase). However, our results did not unveil significant differences in the percentage of cells in the active phases of the cell cycle following stimulation with NRG1β (Figure 3a). To better evaluate cell proliferation upon administration of NRG1β, we then employed the Livecyte^®^ Cell Analysis System, which provides high contrast time-lapse videos with automated live cell analysis. To this end, MCF10A cells were treated with medium containing NRG1β (10 ng/mL) or with control medium, and then their number and morphological features were followed over time up to 72 h. This analysis did not show significant differences in cell number upon administration of NRG1β (Figure 3b and Supplementary Figure S4). We thus concluded that NRG1β does not play a role in the proliferation of MCF10A cells. After stimulation with NRG1β, we also did not observe significant differences in the instantaneous velocity of cells up to 12 h (Figure 3c). Moreover, no major differences in random versus directional migration were observed, as evidenced by similar cell displacement (Figure 3d) and confinement ratio (Figure 3e,f) at different time points (6 and 12 h). Overall, our data suggest that the NRG1β/ERBB3/ERBB2 axis does not play a role in cell proliferation or motility of basal-like breast cells cultured in monolayer conditions.

### 3.3. Administration of NRG1β Does Not Significantly Impact Cell Differentiation in Basal-like/Triple-Negative Breast Cells

**Figure 4:**
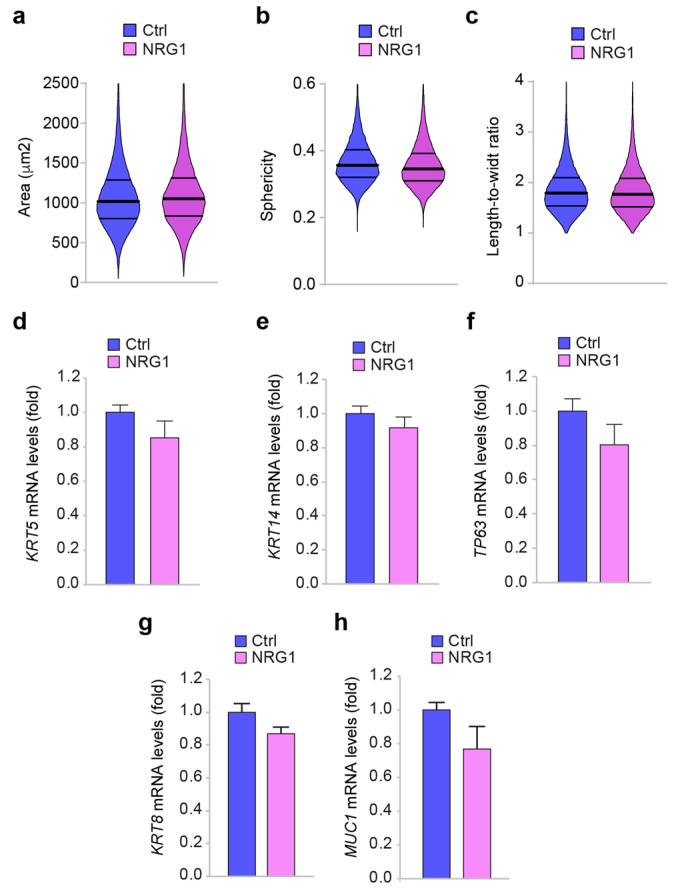
Administration of NRG1β does not influence morphological parameters and the expression of basal/myoepithelial and luminal differentiation markers in basal-like/triple-negative breast cells. (**a**–**c**) Analysis of morphological parameters, namely (**a**) cell size, (**b**) sphericity, and (**c**) length-to-width ratio in MCF10A cells cultured in vitro and treated with/without NRG1β (10 ng/mL) for 72 h; (**d**,**e**) mRNA expression levels of myoepithelial/basal markers cytokeratin 5/6 (**d**) *KRT5* and cytokeratin 14 (**e**) *KRT14* in MCF10A cells cultured in vitro and treated with/without NRG1β (10 ng/mL); (**f**) mRNA expression levels of *TP63* gene in MCF10A cells cultured in vitro and treated with/without NRG1β (10 ng/mL); (**g**,**h**) mRNA expression levels of luminal markers cytokeratin 8 (**g**) *KRT8*, and mucin1 (**h**) *MUC1* in MCF10A cells cultured in vitro and treated with/without NRG1β (10 ng/mL). In all panels, numerical data are presented as the mean (error bars show s.e.m.).

Undifferentiated or poorly differentiated cancer cells tend to grow and spread at a faster rate than well-differentiated cancer cells. We thus analyzed if ERBB3 activation by NRG1β may reduce basal-like breast cell differentiation. Our initial Livecyte analysis of morphological parameters of NRG1β-treated versus control MCF10A cells showed minimal and not statistically significant variations in terms of cell size (Figure 4a), sphericity (Figure 4b), and length-to-width ratio (Figure 4c).

The mammary epithelium consists of differentiated cell types organized into two cell layers, an inner layer of luminal (ductal and alveolar) epithelial cells surrounded by an outer layer of myoepithelial cells in direct contact with the basement membrane [39,40] (reviewed in [41,42,43,44]). Myoepithelial and luminal cells typically express different sets of keratins. In particular, myoepithelial cells express cytokeratin 5/6 (*KRT5*), cytokeratin 14 (*KRT14*), and cytokeratin 17 (*KRT17*), whereas luminal cells express cytokeratin 8 (*KRT8*), cytokeratin 18 (*KRT18*), and cytokeratin 7 (*KRT7*) [39,40] (reviewed in [42,43,44,45]). Basal-like breast cancer is defined by the expression of genes abundantly expressed by basal myoepithelial cells [16,17,46]. Accordingly, mRNA expression of myoepithelial cytokeratins (*KRT5, KRT14*, and *KRT17*) was upregulated in our datasets of primary basal-like tumors (Supplementary Figure S5a–c) and cell lines (Supplementary Figure S6a–c). Similarly, triple-negative cell lines also expressed higher mRNA levels of these keratins (Supplementary Figure S6d–f). Furthermore, high levels of expression of these basal/myoepithelial keratins were observed in MCF10A cells (Supplementary Figure S6g–i), again confirming these cells as a suitable basal-like/triple-negative model.

To evaluate the potential impact of the NRG1β/ERBB3/ERBB2 axis on basal/myoepithelial cell differentiation, we analyzed the expression of basal markers in MCF10A cells exposed to NRG1β. Interestingly, administration of NRG1β to MCF10A did not induce significant changes in KRT5 and KRT14 mRNA expression (Figure 4d,e). Another proposed marker for myoepithelial/basal cells and basal-like tumors is p63 [40] (reviewed in [42]). Surprisingly, in our meta-analyses, the expression of p63 gene (*TP63*) was significantly increased neither in basal-like tumors (Supplementary Figure S7a) nor in basal or triple-negative lines (Supplementary Figure S7b,c). Nevertheless, *TP63* mRNA was highly expressed in MCF10A cells (Supplementary Figure S7d) and was not significantly regulated by exposure to NRG1β (Figure 4f). On the other hand, NRG1β treatment did not influence the mRNA expression of luminal markers, such as cytokeratin 8 (*KRT8*) and mucin 1 (*MUC1*) (Figure 4g,h).

Overall, our data suggest that cell differentiation of basal-like/triple-negative cells is not significantly regulated by the NRG1β/ERBB3/ERBB2 axis.

### 3.4. NRG1β/ERBB3/ERBB2 Robustly Promotes Anchorage-Independent Cell Growth of Basal-like Breast Cells

**Figure 5:**
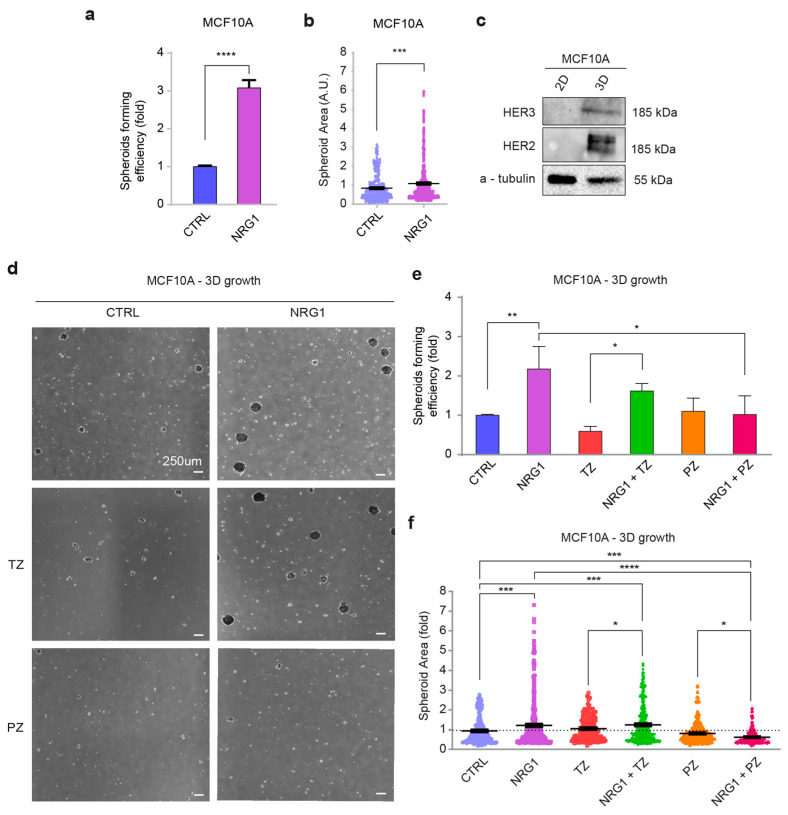
Administration of NRG1β promotes the anchorage-independent growth of basal-like/triple-negative breast cells. (**a**,**b**) Spheroid forming efficiency and growth (size) of MCF10A cells upon stimulation with NRG1β. MCF10A cells were seeded in ultra-low attachment plates and stimulated with/without NRG1β (10 ng/mL) every 3 days for up to 14 days. Spheroid number (spheroid forming efficiency) and size are provided in (**a**,**b**), respectively. (**c**) Western blot analysis of ERBB3 and ERBB2 protein levels in lysates of MCF10A cells cultured in monolayer (2D) and non-adherent conditions (3D). (**d**–**f**) 3D cell growth of MCF10A cells upon stimulation with NRG1β and simultaneous inhibition of ERBB2 by trastuzumab (TZ) or pertuzumab (PZ). MCF10A cells were seeded in ultra-low attachment plates and stimulated with/without NRG1β (10 ng/mL), alone or in combination with trastuzumab (10 µg/mL) or pertuzumab (10 µg/mL). Stimulations were repeated every 3 days up to 14 days. Representative pictures are provided in (**d**). Spheroid number (spheroid forming efficiency) and size (spheroid growth) are provided in (**e**,**f**), respectively. In all panels, numerical data are normalized to control cells and presented as the mean (error bars show s.e.m.); statistical significance was determined using Student’s *t*-test in (**a**,**b**), and one-way ANOVA followed by Tukey’s test in (**e**,**f**); * *p* < 0.05, ** *p* < 0.01, *** *p* < 0.001, and **** *p* < 0.0001.

Our clinical data showed a correlation between higher *ERBB3* mRNA expression levels and shorter relapse-free survival in basal-like breast cancer patients. Since our initial analyses did not unveil significant effects induced by NRG1β on cell proliferation, differentiation, and migration of basal-like/triple-negative cells, we decided to test the role of the NRG1β/ERBB3/ERBB2 axis in non-adherent conditions. To this aim, cells were seeded in ultra-low attachment plates, which feature a covalently bound hydrogel layer that effectively inhibits cellular attachment. At first, we measured cell survival in response to NRG1β stimulation by flow cytometry analysis of Annexin V/propidium iodide (PI) staining. In particular, Annexin V binds to cells in early apoptosis, whereas the PI binds to DNA into the nucleus in the late stages of cellular apoptosis and to necrotic cells. However, no appreciable effects upon stimulation with NRG1β were observed at all analyzed time points, namely 24, 48, and 72 h after the stimulation (Supplementary Figure S8a–c). Afterward, we evaluated the impact of the NRG1β/ERBB3/ERBB2 axis in promoting cell growth in non-adherent conditions. To this end, cells were seeded in ultra-low attachment plates and every three days were treated with NRG1β, by replacing half of the medium. The number and size of spheroids, namely three-dimensional (3D) cell cultures that arrange themselves during proliferation into sphere-like formations, were then measured after 14 days in culture. Strikingly, in contrast to 2D monolayer cell condition, NRG1β promoted an increase in both spheroid number and size (Figure 5a,b). Interestingly, MCF10A cells cultured in 3D compared to 2D conditions strongly upregulated ERBB3 and ERBB2 protein levels (Figure 5c). These data suggest a role for ERBB3 and ERBB2 in mediating the anchorage-independent growth induced by NRG1β in basal-like/triple-negative breast cells. Next, we sought to neutralize ERBB2, by employing two anti-ERBB2 agents, namely trastuzumab and pertuzumab, currently used in clinics. Trastuzumab is used to treat HER2-positive breast cancer patients in metastatic and adjuvant settings (reviewed in [1,47]). It is a humanized monoclonal antibody that binds to the extracellular domain IV of HER2, strongly inhibiting its ligand-independent activation, which has been reported to mainly occur when ERBB2 is overexpressed [48,49] (reviewed in [1,47,50]). Pertuzumab has shown antitumor activity in both the metastatic and the neoadjuvant settings of HER2-positive breast cancer patients and has been more recently approved as adjuvant therapy. It is a humanized monoclonal antibody that binds to the extracellular domain II of HER2, essential for dimerization (reviewed in [47,50]). Thus, pertuzumab was suggested to efficiently inhibit ligand-dependent HER2 dimerization. In contrast, trastuzumab was reported to be less effective in the presence of ERBB ligands [51] (reviewed in [47]). Co-administration of trastuzumab or pertuzumab was therefore used to evaluate the contribution of ERBB2 to NRG1β signaling in anchorage-independent growth (Figure 5d). In our experiments, administration of trastuzumab was not efficient in reducing the increase in spheroids’ formation driven by NRG1β, even if a trend toward a reduction in spheroids’ formation was observed when administered alone (Figure 5e). Furthermore, trastuzumab, alone or in combination with NRG1β, was unable to reduce spheroid’s size (Figure 5f). In contrast, pertuzumab completely suppressed the action of NRG1β, despite not showing any effect on spheroids’ number or size when administered alone (Figure 5d–f).

**Figure 6:**
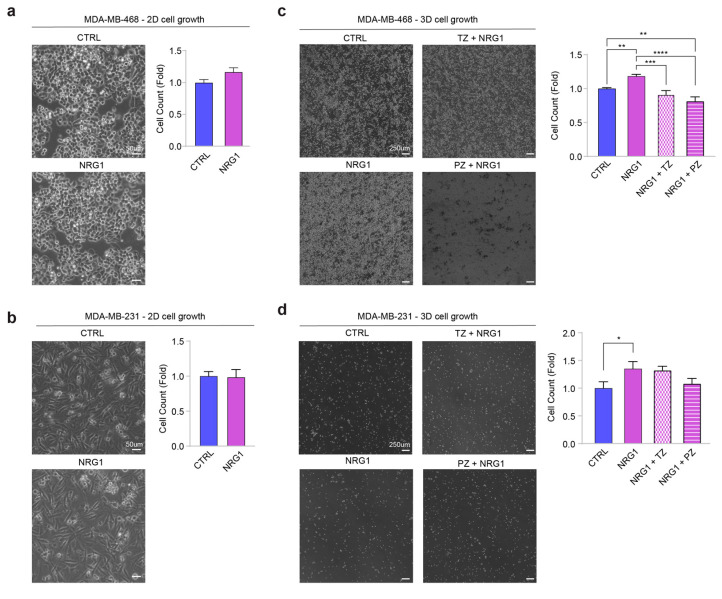
Administration of NRG1β promotes the anchorage-independent growth of basal-like/triple-negative breast cancer cells. (**a**,**b**) Anchorage-dependent cell proliferation of MDA-MB-468 and MDA-MB-231 cells upon stimulation with NRG1β. MDA-MB-468 and MDA-MB-231 cells were seeded in adherent conditions and stimulated with/without NRG1β (10 ng/mL) for 72 h. Cell count for MDA-MB-468 and MDA-MB-231 is provided in (**a**) and (**b**), respectively. (**c**,**d**) Anchorage-independent cell growth of MDA-MB-468 and MDA-MB-231 cells upon stimulation with NRG1β and simultaneous inhibition of ERBB2 by trastuzumab (TZ) or pertuzumab (PZ). MDA-MB-468 and MDA-MB-231 cells were seeded in ultra-low attachment plates and stimulated with/without NRG1β (10 ng/mL), alone or in combination with trastuzumab (10 µg/mL) or pertuzumab (10 µg/mL). Stimulations were repeated every 3 days for up to 14 days. Representative pictures and cell count for MDA-MB-468 and MDA-MB-231 are provided in (**c**) and (**d**), respectively. In all panels, numerical data are normalized to control cells and presented as mean (error bars show s.e.m.); statistical significance was determined using Student’s *t*-test in (**a**,**b**), and one-way ANOVA followed by Tukey’s test in (**c**,**d**); * *p* < 0.05, ** *p* < 0.01, *** *p* < 0.001, and **** *p* < 0.0001.

Since MCF10A cells are considered a quasi-normal basal-like/triple-negative cellular model, we decided to validate our findings on basal-like/triple-negative breast cancer cells. To this end, we employed MDA-MB-468 and MDA-MB-231 cells. In line with the data obtained on MCF10A cells, administration of NRG1β did not significantly induce cell proliferation when MDA-MB-231 and MDA-MB-468 cells were cultured in adhesion (Figure 6a,b); however, it promoted their cell growth when cultured in anchorage-independent conditions (Figure 6c,d). Interestingly, in MDA-MB-468, this process was inhibited by both trastuzumab and pertuzumab, whereas in MDA-MB-231, we observed a trend of reduction only with pertuzumab (Figure 6c,d).

Overall, our data unveil that the NRG1β/ERBB3/ERRB2 axis promotes cell growth in suspension of basal-like/triple-negative breast cancer cells and that this process is efficiently prevented by the administration of anti-ERBB2 agents, in particular, pertuzumab.

### 3.5. Pertuzumab Efficiently Blocks NRG1β-Induced Cell Growth in Non-Adherent Conditions in HER2-Overexpressing Basal-like Breast Cells

**Figure 7:**
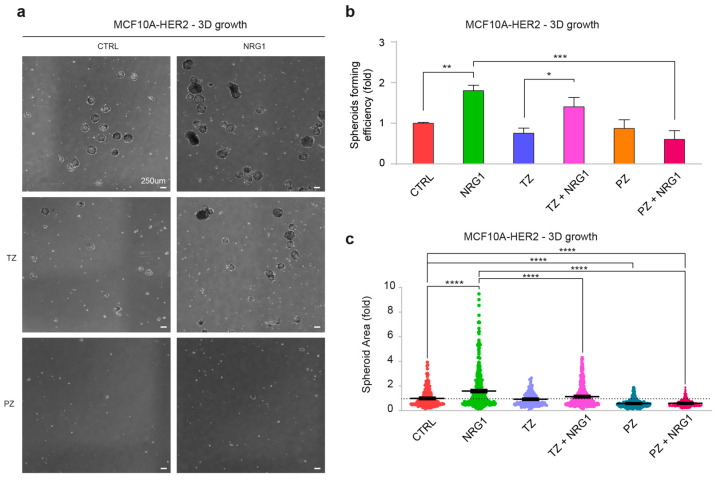
Administration of NRG1β promotes growth in non-adherent conditions in basal-like/triple-negative breast cells engineered with HER2 overexpression. (**a**–**c**) Spheroid forming efficiency and growth (size) of HER2-overexpressing MCF10A (MCF10-HER2) upon stimulation with NRG1β. MCF10A cells were seeded in ultra-low attachment plates and stimulated with/without NRG1β (10 ng/mL), trastuzumab (TZ, 10 µg/mL) or pertuzumab (PZ, 10 µg/mL) every 3 days up to 14 days. Representative pictures of spheroids are provided in (**a**); spheroids number (spheroid forming efficiency) and size are provided in (**b**,**c**), respectively. In all panels, numerical data are normalized to control cells and presented as mean (error bars show s.e.m.); statistical significance was determined using one-way ANOVA followed by Tukey’s test in (**b**,**c**); * *p* < 0.05, ** *p* < 0.01, *** *p* < 0.001, and **** *p* < 0.0001.

About 25% of triple-negative breast cancers with HER2-negative expression in the primary tumor convert to HER2-low expression in the recurrent tumor, maintaining the same cancer phenotype [52]. Our study suggests that the loss of cell adhesion may contribute to ERBB2 upregulation. To evaluate the role of the NRG1β/ERBB3/ERBB2 axis in basal-like cells with increased expression of ERBB2, we also analyzed previously generated MCF10A cells stably overexpressing ERBB2 (MCF10A-HER2) [31,32]. Administration of NRG1β to MCF10A-HER2 induced an increase in spheroid’s number and size compared to untreated control cells, which was significantly prevented by pertuzumab, but not by trastuzumab co-administration (Figure 7a–c). Overall, our data suggest that pertuzumab efficiently inhibits the growth of basal-like breast cells induced by NRG1β, even when ERBB2 is upregulated.

## 4. Discussion

In this study, we report an inverse correlation between *ERBB3* mRNA expression levels and relapse-free survival in basal-like breast cancer patients. Of note, although ERBB3 role in breast cancer progression is well established, our work suggests its specific involvement in basal-like/triple-negative breast cancer cells. By employing the growth factor neuregulin 1β (NRG1β), we analyzed the impact of ERBB3 signaling activation on multiple phenotypes in a basal-like quasi-normal breast cellular model (MCF10A cells). Notably, our data demonstrate that NRG1β administration strongly increases basal-like/triple-negative cell growth as spheroids in non-adherent conditions. Importantly, the ability to grow in anchorage-independent conditions, thus forming spheroids, is thought to be responsible for metastatic tumor dissemination, which in turn is the cause of death in more than 90% of human cancer patients [53]. Metastasis is indeed a multistep process in which cancer cells invade the basement membrane, dissociate from primary sites, intravasate in the vascular or lymphatic system, survive to the circulation, extravasate from the vasculature to secondary tissue, and finally proliferate in distant organs. As a barrier to metastasis, cells normally undergo an apoptotic process known as “anoikis,” a form of cell death consequent to loss of adhesion with the extracellular matrix or with neighboring cells. Cancer cells acquire anoikis resistance to survive after detachment from the primary sites and travel through the circulatory and lymphatic systems to disseminate throughout the body. Intriguingly, NRG1β administration was unable to support cell survival of basal-like breast cells cultured in suspension. Thus, our data suggest that NRG1β may specifically support the development and growth of metastasis in basal-like breast cancer patients.

In our study, the activation of the NRG1β/ERBB3/ERBB2 axis in basal-like/triple-negative breast cells cultured in monolayer did not induce cell proliferation and motility, and did not significantly impact cell differentiation. Nevertheless, we cannot exclude the possibility that chronic exposure to NRG1β would impact these phenotypes in the long term. In addition, our study did not evaluate the potential role of NRG1 directly produced by cancer cells. In this regard, silencing of NRG1 in triple-negative cellular models has been very recently documented to reduce cell migration [54], further supporting a role for NRG1 signaling in basal-like/triple-negative cancers. Previously, NRG1 administration to MCF10A cells engineered with ERBB2 overexpression was demonstrated to induce an invasive phenotype with protruded arms when cultured in 3D conditions [32]. Stimulation with neuregulin 1 after combinatorial overexpression of wild-type ERBB3 and ERBB2 has been shown to promote anchorage-independent growth in MCF10A cells [55]. Moreover, overexpression of active ERBB3 mutants and wild-type ERBB2 promoted similar effects in a ligand-independent manner [6]. Our data in MCF10A cells suggest that NRG1β is more effective in supporting 3D compared to 2D cell growth, due to an upregulation in ERBB3 and ERBB2 receptor abundance when cells are grown in anchorage-independent conditions. This is particularly interesting considering that it has been reported that about 36% of triple-negative breast cancers can switch from HER2-negative to HER2-low expression, whereas about 5% can switch from HER2-negative to HER2-positive [52]. Our work suggests that the loss of cell adhesion, which occurs after intravasation in the vascular or lymphatic system during the metastatic process, may contribute to these transitions. Nevertheless, preliminary analyses did not confirm this upregulation in MDA-MB-468 and MDA-MB-231 when cultured in anchorage-independent conditions (data not shown), suggesting that this mechanism may be influenced by specific genetic alterations occurring in cancer cells.

The role of ERBB2 in cancer progression is well established (reviewed in [1,5]) and targeting ERBB2 through specific monoclonal antibodies, such as trastuzumab and pertuzumab, represents the gold standard to treat HER2-positive breast cancer patients in clinics (reviewed in [1]). So far, only cancer patients with strong HER2 expression are treated with anti-HER2 drugs. Indeed, in common clinical practice, the treatment-decision process is driven by the dichotomization in HER2-positive vs. negative, with HER2-positive defined by a score of 3+ HER2 protein overexpression in IHC analysis and/or HER2 gene amplification in an ISH assay. However, HER2-negative breast cancers are characterized by a wide spectrum of HER2 expression levels, with about half of breast cancers classified as HER2-negative showing low HER2 expression [52]. Notably, preliminary results from early phase clinical trials testing trastuzumab-drug conjugates in advanced breast cancer patients harboring HER2-low expression were promising [56,57] (reviewed in [58]). On the other hand, a phase II trial showed disappointing tumor responses upon pertuzumab administration alone to HER2-negative or HER2-low breast cancer patients [59] (reviewed in [58]). Based on our data, we here suggest targeting the NRG1/ERBB3/ERBB2 signaling axis in basal-like/triple-negative breast tumors. The oncogenic role of ERBB2 has been poorly investigated in basal-like/triple-negative breast cancers owing to the low expression of this receptor in these breast cancer subtypes. Despite that, quite recently the canonical ERBB2 isoform and an ERBB2 variant located in the nucleus were suggested to drive triple-negative breast cancer growth [60]. In our study, administration of pertuzumab was able to suppress spheroid’s forming efficiency and growth driven by NRG1β stimulation in all analyzed basal-like/triple-negative cellular models, whereas trastuzumab was effective only in MDA-MB-468 cells. Even if the differences in trastuzumab response in other basal-like cell lines deserve further investigations, our data are mostly in line with recent literature suggesting that pertuzumab more efficiently blocks ligand-induced ERBB2 activation [51] (reviewed in [47]). We thus suggest the specific administration of pertuzumab to basal-like breast cancer patients, which, to our knowledge, has not been yet tested. Because dual HER2-targeted therapy has been reported to improve overall survival and progression-free survival compared to single HER2-targeted therapy [61] (reviewed in [62]), we also suggest evaluation of this combinatorial therapy as a treatment for basal-like/triple-negative breast cancer patients.

## 5. Conclusions

Our study unveils a link between high ERBB3 expression and poor relapse-free patients’ survival in basal-like breast cancer patients. Despite ERBB3 expression in basal-like/triple-negative breast cancer appearing to be low, our study suggests that the NRG1β/ERBB3/ERBB2 axis in basal-like/triple-negative breast cancer patients may support tumor cell dissemination by promoting anchorage-independent cell growth. Importantly, NRG1β-induced anchorage-independent growth of basal-like/triple-negative breast cancer cells could be inhibited by the administration of anti-HER2 agents, in particular, pertuzumab. The neutralization of the NRG1β/ERBB3/ERBB2 axis deserves further studies as a therapeutic strategy for basal-like/triple-negative breast cancers, which today have very limited treatment opportunities.

## Data Availability

The data presented in this study are available in the Supplementary Material.

## Supplementary Materials

The following supporting information can be downloaded at: https://www.mdpi.com/article/10.3390/cancers14071603/s1, Figure S1. NRG1 expression in normal breast tissue and in breast cancer patients stratified for clinical and molecular subtypes. (a) Box plot of NRG1 gene expression in normal breast tissues and in breast cancer patients stratified for clinical subtypes (*n* = 833 patients); (b) Box plot of NRG1 gene expression in breast cancer patients stratified for molecular subtypes (PAM50) (*n* = 9672 patients); Figure S2. mRNA expression of *ERBB3* and *NRG1* in breast cell lines. (a–f) mRNAs expression of (a–c) *ERBB3* and (d–f) *NRG1* in 51 breast cancer cell lines stratified for (a,b,d,e) molecular clusters and (c,f) clinical subtypes; Figure S3. mRNA expression of *ERBB2*, Estrogen Receptor (*ESR1*), and Progesterone Receptor (*PR*) in breast cell lines. (a–i) mRNAs expression of (a–c) *ERBB2*, (d–f) *ESR1* and (g–i) *PR* in breast cancer cell lines stratified for (a,c,d,f,g,i) molecular clusters and (b,e,h) clinical subtypes; Figure S4. Time-lapse analysis of basal-like breast cells treated in vitro with Neuregulin 1 (NRG1). Time-lapse representative images of MCF10A cells treated with/without NRG1β (10 ng/mL) up to 72 h (1 h, 6 h, 12 h, 24 h, 36 h, 48 h and 72 h); Figure S5. Expression of basal/myoepithelial markers in breast cancer patients stratified for molecular subtypes. (a–c) mRNA levels of (a) cytokeratin 5 (*n* = 9353 patients), (b) cytokeratin 14 (*n* = 9727 patients), and (c) cytokeratin 17 (*n* = 9727 patients) in breast cancer patients stratified for molecular subtypes (PAM 50); Figure S6. Expression of basal/myoepithelial markers in breast cell lines stratified for molecular cluster and clinical subtypes. (a–i) mRNAs expression of (a,d,g) Keratin 5 (*KRT5*), (b,e,h) Keratin 14 (*KRT14)*, (c,f,i) Keratin 17 (*KRT17*) in breast cancer cell lines stratified for (a–c,g–i) molecular clusters and (d–f) clinical subtypes; Figure S7. Expression of *p63* in breast cancer patients and cell lines stratified for molecular cluster and clinical subtypes. (a) *TP63* mRNA levels in breast cancer patients stratified for molecular subtypes (PAM 50); (b–d) mRNAs expression of *TP63* in breast cancer cell lines stratified for (b, d) molecular clusters and (c) clinical subtypes; Figure S8. Administration of NRG1β does not affect the survival of basal-like/triple-negative breast cells under anchorage-independent conditions. (a–c) Flow cytometry analysis of the percentage of early (lower right quadrant) and late (upper right quadrant) apoptosis in MCF10A control cells and treated with NRG1β (10 ng/mL) after (a) 24, (b) 48, and (c) 72 h, respectively. In the flow cytometry profile X-axis and Y-axis exhibit the APC-Annexin V staining and the Propidium Iodide (PI) staining, respectively. Graphs show the percentage of living, early, and apoptotic/necrotic cells at 24, 48, and 72 h; Figure S9. The original western blot figures; Table S1. Sequences of the primers used in this study to analyze mRNA levels by Reat Time PCR.
