# Supplementary material for: NRG1/ERBB3/ERBB2 Axis Triggers Anchorage-Independent Growth of Basal-like/Triple-Negative Breast Cancer Cells
Source: Cancers (Basel). 2022 Mar 22;14(7):1603. doi: 10.3390/cancers14071603 (PMC8997077; doi:10.3390/cancers14071603)
Supplement: Supplementary file 1 [file cancers-14-01603-s001.zip › cancers-1605632 supplementary.pdf]

cancers

MDPI

Supplementary material

NRG1/ERBB3/ERBB2 Axis Triggers Anchorage-Independent Growth of Basal-like/Triple-Negative Breast Cancer Cells

Carmen Miano, Alessandra Morselli, Francesca Pontis, Chiara Bongiovanni, Francesca Sacchi, Silvia Da Pra, Donatella Romaniello, Riccardo Tassinari, Michela Sgarzi, Elvira Pantano, Carlo Ventura, Mattia Lauriola and Gabriele D'Uva

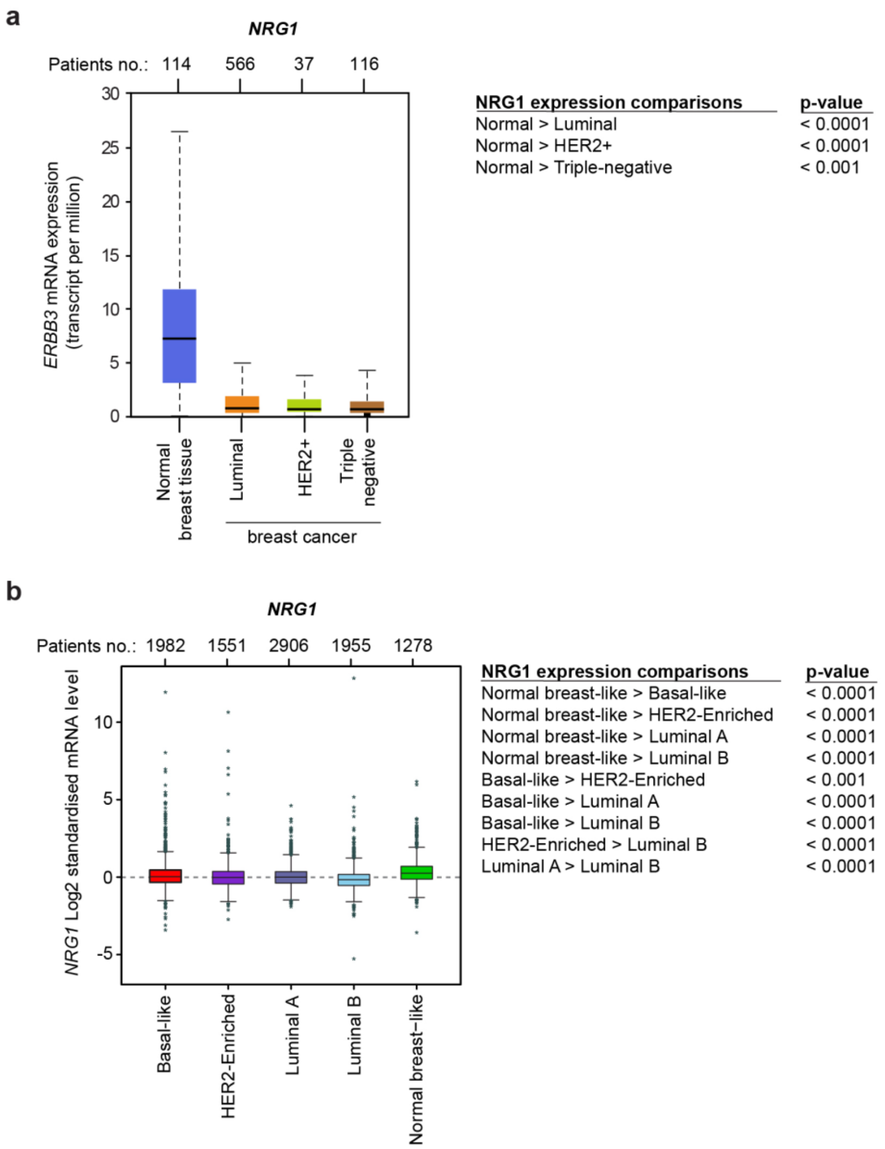

Figure S1. NRG1 expression in normal breast tissue and in breast cancer patients stratified for clinical and molecular subtypes. (a) Box plot of *NRG1* gene expression in normal breast tissues and in breast cancer patients stratified for clinical subtypes (n = 833 patients); (b) Box plot of *NRG1* gene expression in breast cancer patients stratified for molecular subtypes (PAM50) (n = 9672 patients).

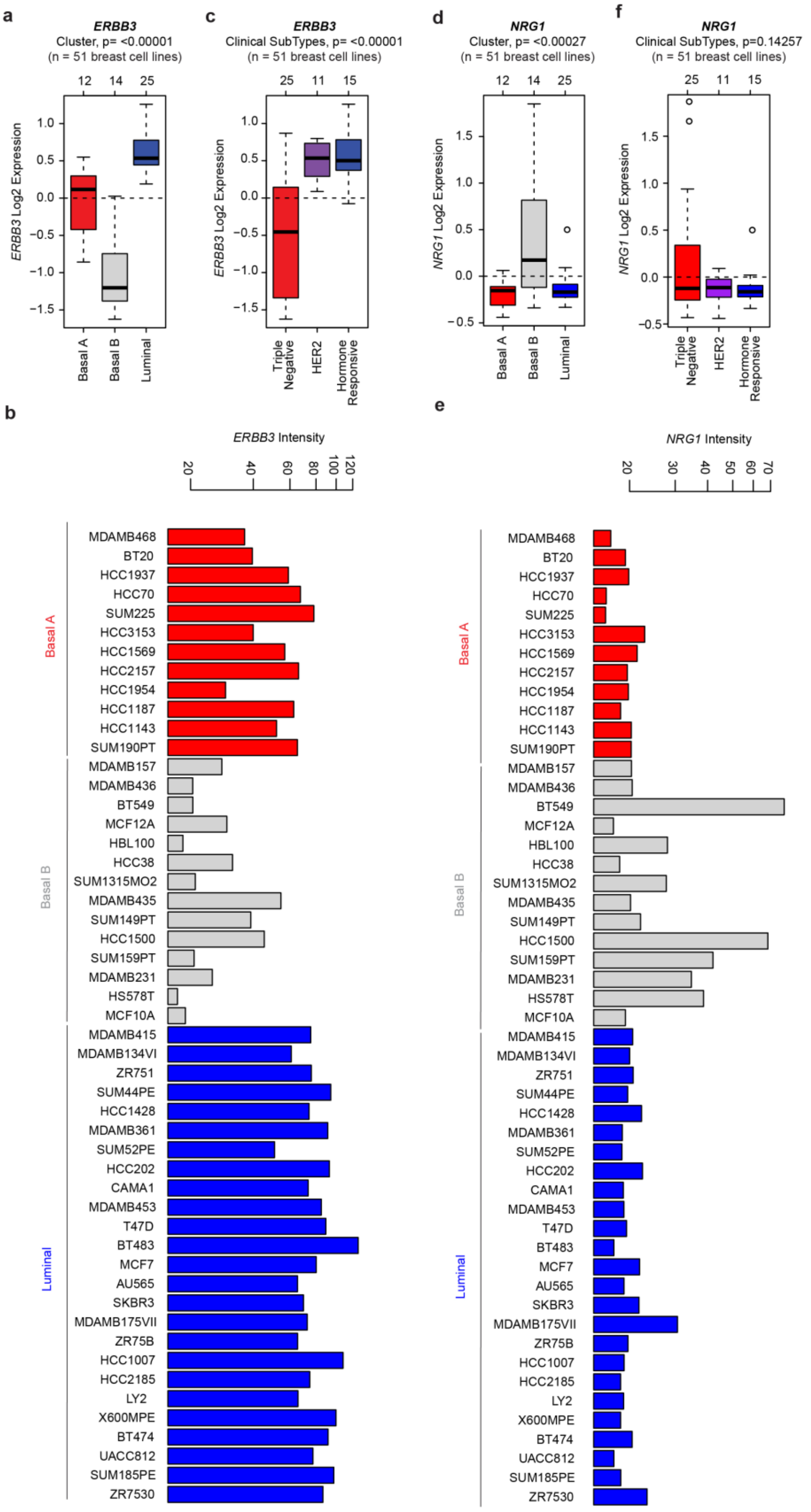

Figure S2. mRNA expression of ERBB3 and NRG1 in breast cell lines. (a-f) mRNAs expression of (**a-c**) *ERBB3* and (**d-f**) *NRG1* in 51 breast cancer cell lines stratified for (**a, b, d, e**) molecular clusters and (**c, f**) clinical subtypes.

.

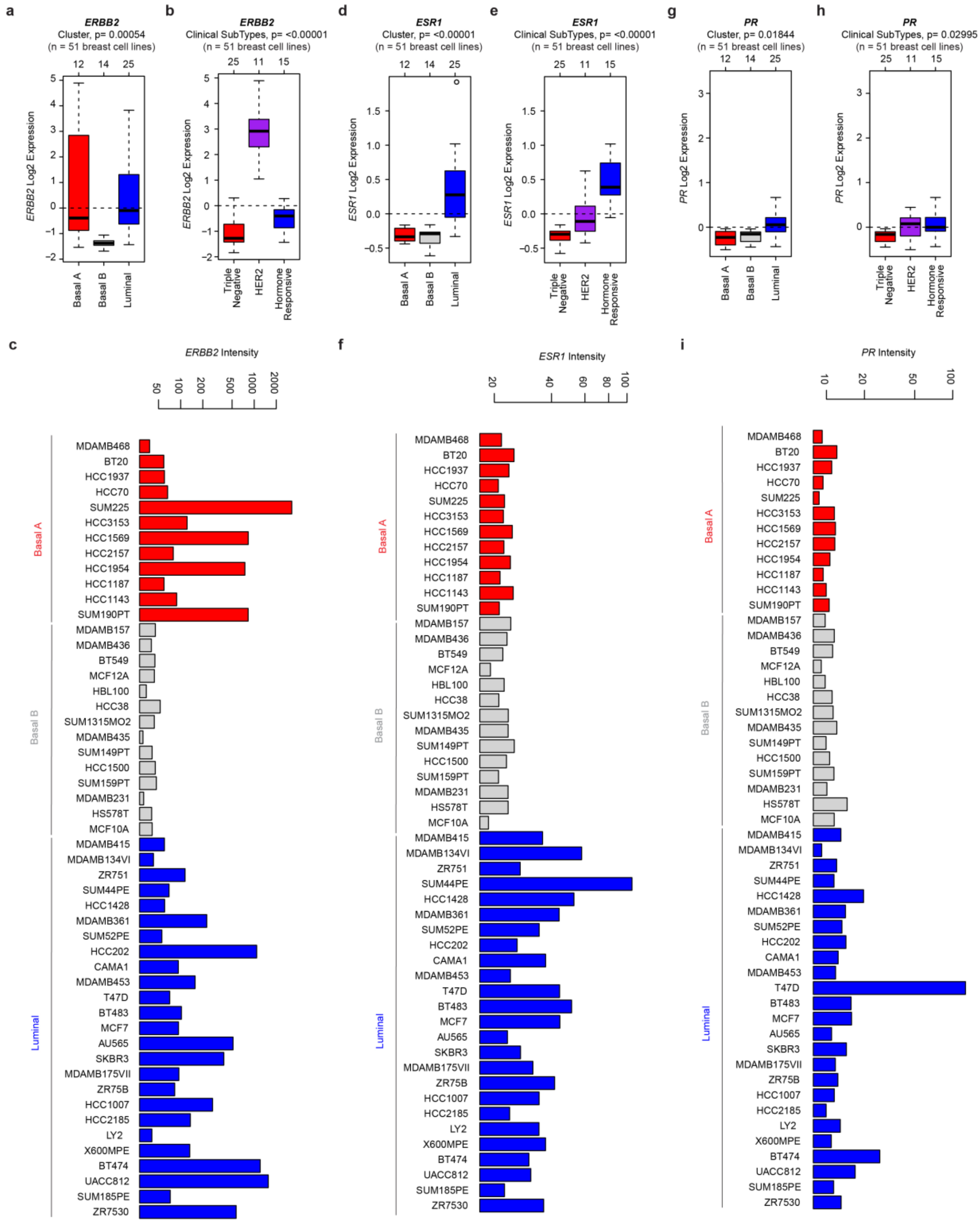

Figure S3. mRNA expression of ERBB2, Estrogen Receptor (ESR1), and Progesterone Receptor (PR) in breast cell lines. (a-i) mRNAs expression of (a-c) *ERBB2*, (d-f) *ESR1 and* (g-i) *PR* in breast cancer cell lines stratified for (a, c, d, f, g, i) molecular clusters and (b,e,h) clinical subtypes.

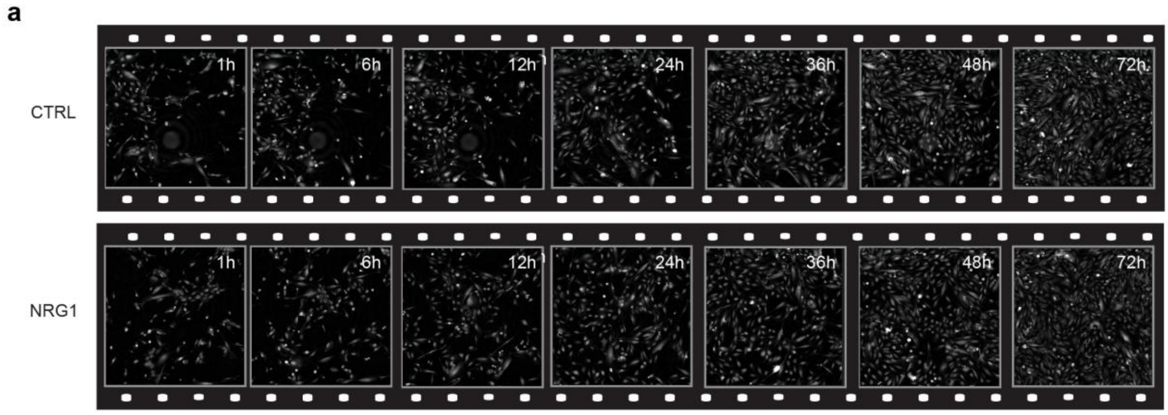

Figure S4. Time-lapse analysis of basal-like breast cells treated *in vitro* with Neuregulin 1 (NRG1). Time-lapse representative images of MCF10A cells treated with/without NRG1β (10 ng/mL) up to 72 hours (1h, 6h, 12h, 24h, 36h, 48h and 72h).

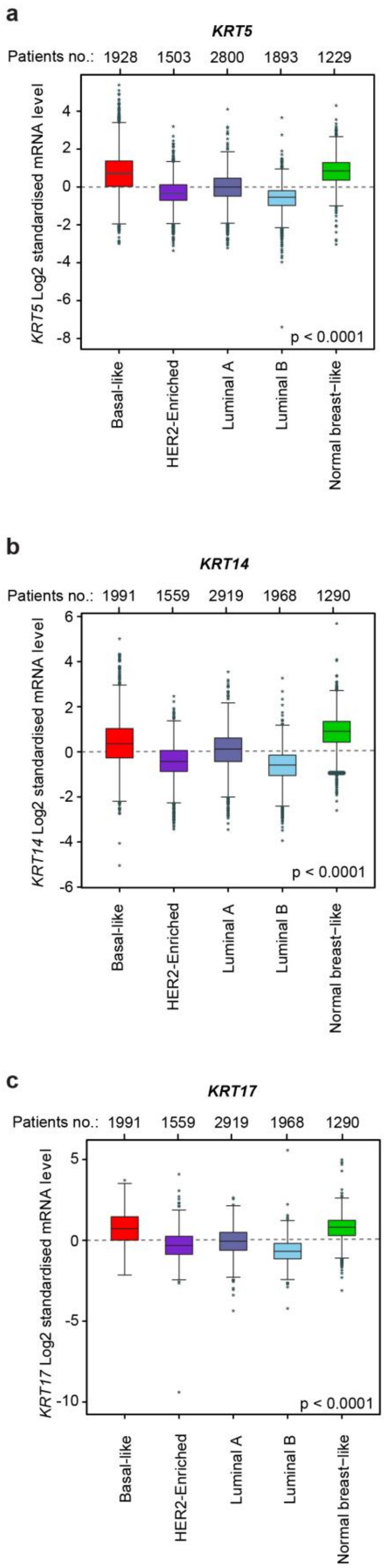

KRT5 expression comparisons	p-value
Basal-like > Luminal A	< 0.0001
Basal-like > HER2-Enriched	< 0.0001
Basal-like > Luminal B	< 0.0001
Normal breast-like > Luminal A	< 0.0001
Normal breast-like > HER2-Enriched	< 0.0001
Normal breast-like > Luminal B	< 0.0001
Luminal A > HER2-Enriched	< 0.0001
Luminal A > Luminal B	< 0.0001
HER2-Enriched > Luminal B	< 0.0001

KRT14 expression comparisons	p-value
Normal breast-like > Basal-like	< 0.0001
Normal breast-like > Luminal A	< 0.0001
Normal breast-like > HER2-Enriched	< 0.0001
Normal breast-like > Luminal B	< 0.0001
Basal-like > Luminal A	< 0.0001
Basal-like > HER2-Enriched	< 0.0001
Basal-like > Luminal B	< 0.0001
Luminal A > HER2-Enriched	< 0.0001
Luminal A > Luminal B	< 0.0001
HER2-Enriched > Luminal B	< 0.0001

KRT17 expression comparisons	p-value
Basal-like > Luminal A	< 0.0001
Basal-like > HER2-Enriched	< 0.0001
Basal-like > Luminal B	< 0.0001
Normal breast-like > Luminal A	< 0.0001
Normal breast-like > HER2-Enriched	< 0.0001
Normal breast-like > Luminal B	< 0.0001
Luminal A > HER2-E	< 0.0001
Luminal A > Luminal B	< 0.0001
HER2-Enriched > Luminal B	< 0.0001

Figure S5. Expression of basal/myoepithelial markers in breast cancer patients stratified for molecular subtypes. (a-c) mRNA levels of (**a**) cytokeratin 5 (n = 9353 patients), (**b**) cytokeratin 14

(n =9727 patients), and (**c**) cytokeratin 17 (n = 9727 patients) in breast cancer patients stratified for molecular subtypes (PAM 50).

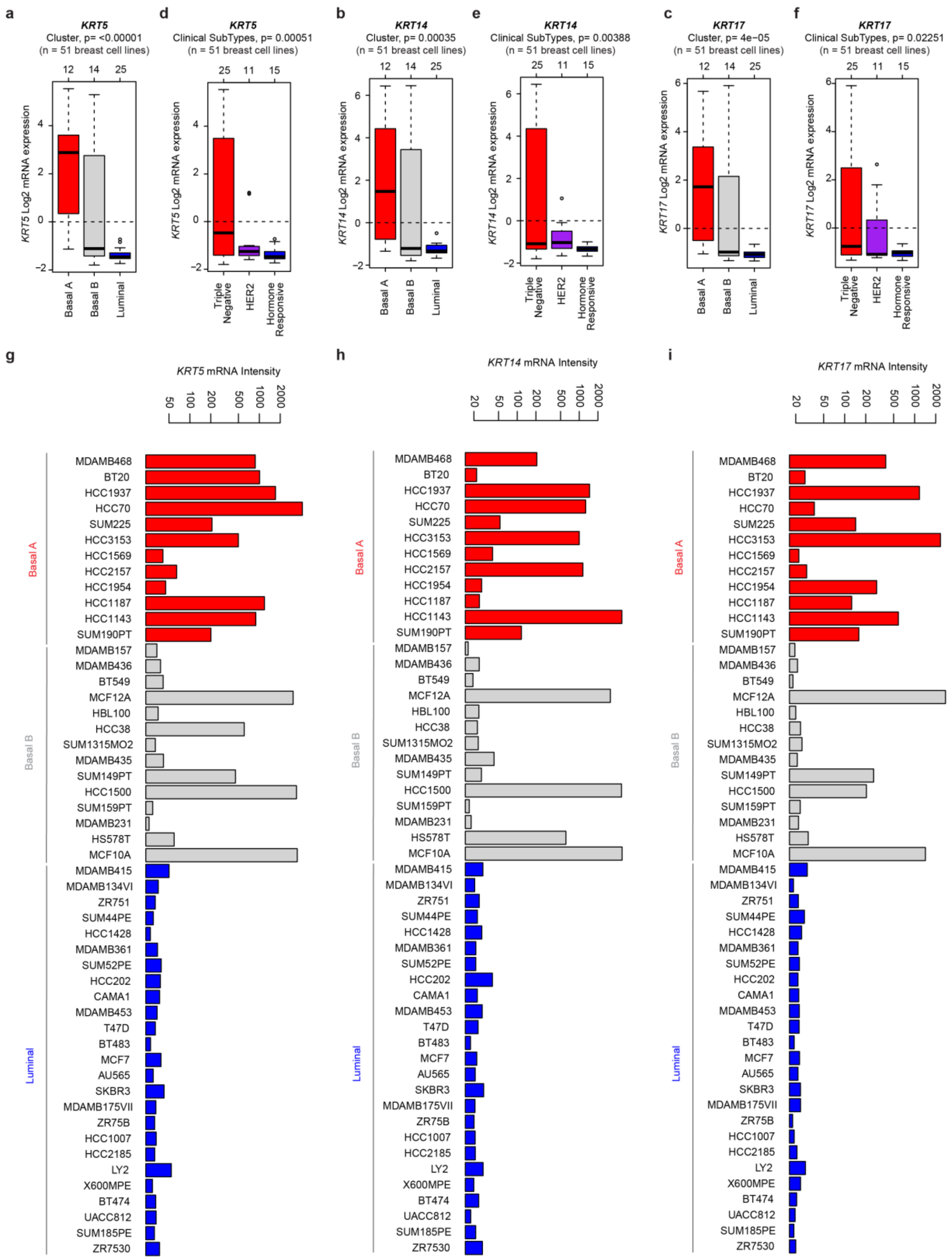

Figure S6. Expression of basal/myoepithelial markers in breast cell lines stratified for molecular cluster and clinical subtypes. (a-i) mRNAs expression of (a,d,g) Keratin 5 (*KRT5*), (b,e,h) Keratin 14 (*KRT14*), (c,f,i) Keratin 17 (*KRT17*) in breast cancer cell lines stratified for (a-c, g-i) molecular clusters and (d-f) clinical subtypes.

.

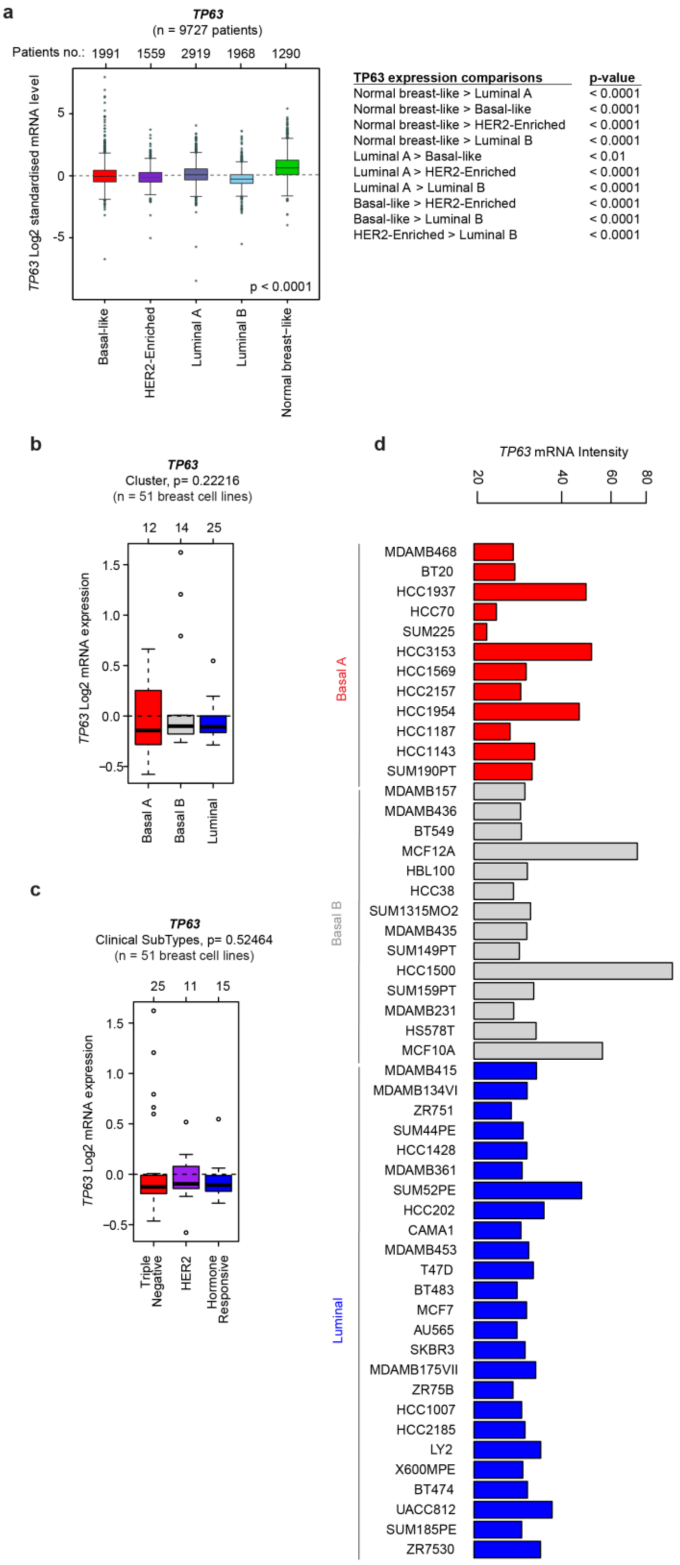

Figure S7. Expression of p63 in breast cancer patients and cell lines stratified for molecular cluster and clinical subtypes. (a) *TP63* mRNA levels in breast cancer patients stratified for molecular subtypes (PAM 50); (b-d) mRNAs expression of *TP63* in breast cancer cell lines stratified for (b, d) molecular clusters and (c) clinical subtypes.

.

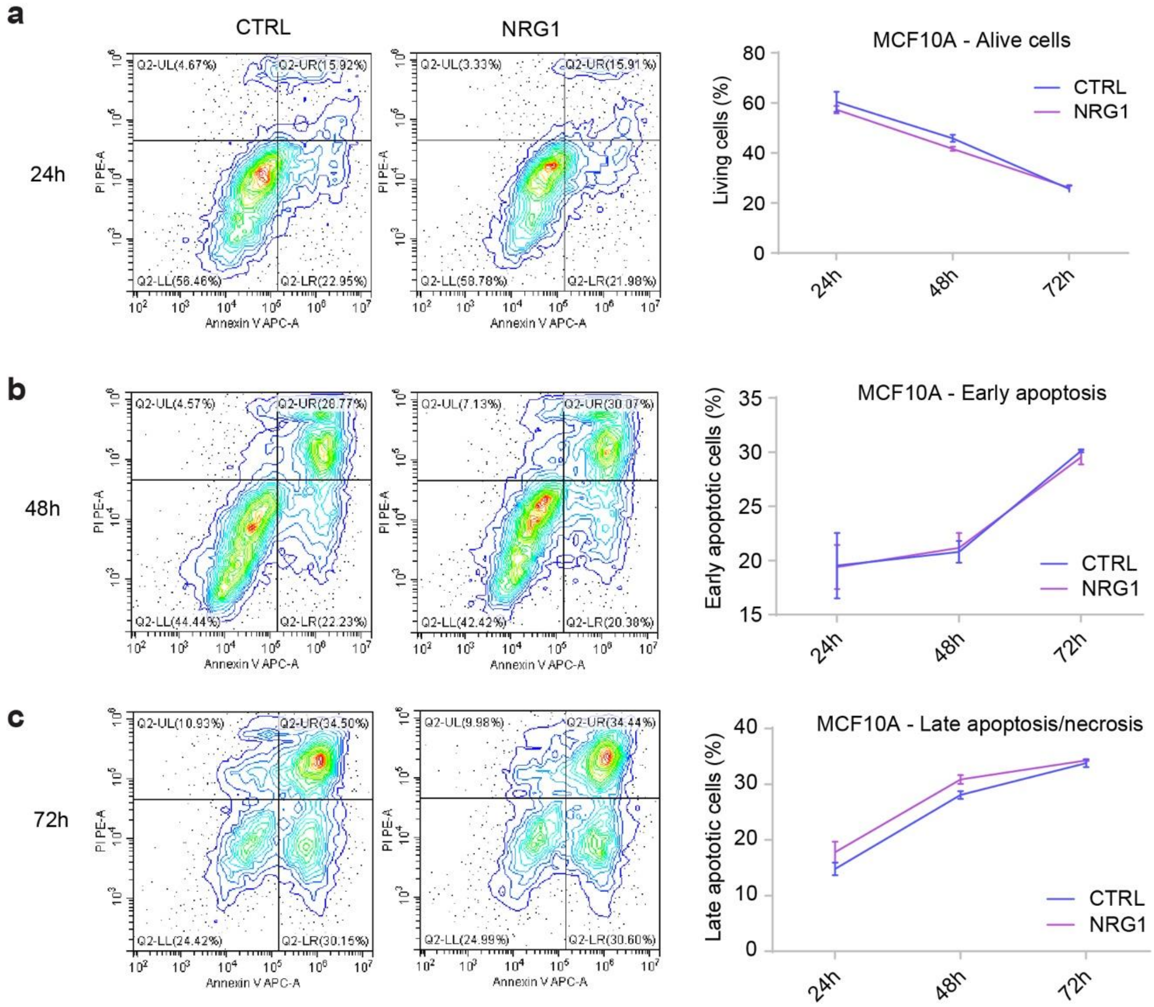

Figure S8. Administration of NRG1β does not affect the survival of basal-like/triple-negative breast cells under anchorage-independent conditions. (a-c) Flow cytometry analysis of the percentage of early (lower right quadrant) and late (upper right quadrant) apoptosis in MCF10A control cells and treated with NRG1β (10 ng / ml) after (a) 24, (b) 48, and (c) 72 hours, respectively. In the flow cytometry profile X-axis and Y-axis exhibit the APC-Annexin V staining and the Propidium Iodide (PI) staining, respectively. Graphs show the percentage of living, early, and apoptotic/necrotic cells at 24, 48, and 72 hours.

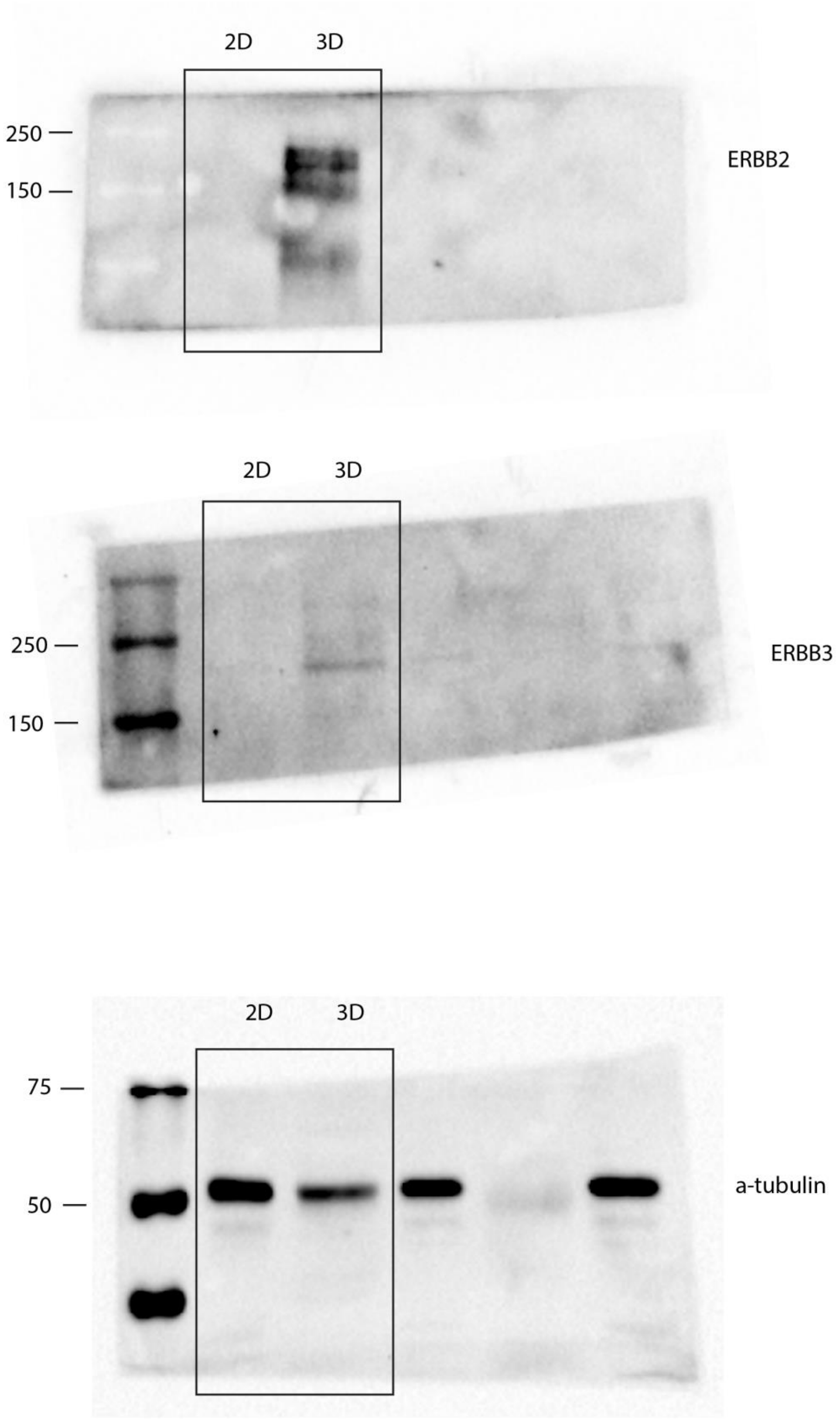

Figure S9. The original western blot figures.

Table S1. Sequences of the primers used in this study to analyze mRNA levels by real-time (rt)PCR.

Gene	Forward primer	Reverse primer
KRT5	AGTTTGTGATGCTGAAGAAG	GTTAATCTCATCCATCAGTGC
KRT14	AGATCAAAGACTACAGTCCC	ACTCTGTCTCATACTTGGTG
TP63	CAGCCTATATGTTCAGTTCAG	CAGTCCATGCTAATCTCAATC
KRT8	ACGAATTTGTCCTCATCAAG	CCGGATCTCCTCTTCATATAG
MUC1	GCCTCTCCAATATTAAGTTCAG	AGATCGTCAGGTTATATCGAG
HPRT1	ATAAGCCAGACTTTGTTGG	ATAGGACTCCAGATGTTTCC
